# 3D Printed Chitosan-Pectin Hydrogels: From Rheological Characterization to Scaffold Development and Assessment

**DOI:** 10.3390/gels7040175

**Published:** 2021-10-21

**Authors:** Iratxe Zarandona, Carlos Bengoechea, Estefanía Álvarez-Castillo, Koro de la Caba, Antonio Guerrero, Pedro Guerrero

**Affiliations:** 1 BIOMAT Research Group, University of the Basque Country (UPV/EHU), Escuela de Ingeniería de Gipuzkoa, Plaza de Europa 1, 20018 Donostia-San Sebastián, Spain; iratxe.zarandona@ehu.eus; 2 Departamento de Ingeniería Química, Universidad de Sevilla, Escuela Politécnica Superior, Calle Virgen de África, 7, 41011 Sevilla, Spain; cbengoechea@us.es (C.B.); malvarez43@us.es (E.Á.-C.); aguerrero@us.es (A.G.); 3 BCMaterials, Basque Center for Materials, Applications and Nanostructures, UPV/EHU Science Park, 48940 Leioa, Spain; 4 Proteinmat Materials SL, Avenida de Tolosa 72, 20018 Donostia-San Sebastián, Spain

**Keywords:** chitosan, pectin, 3D printing, rheology, scaffolds

## Abstract

Chitosan-pectin hydrogels were prepared, and their rheological properties were assessed in order to select the best system to develop scaffolds by 3D printing. Hydrogels showed a weak gel behavior with shear thinning flow properties, caused by the physical interactions formed between both polysaccharides, as observed by FTIR analysis. Since systems with high concentration of pectin showed aggregations, the system composed of 2 wt% chitosan and 2 wt% pectin (CHI2PEC2) was selected for 3D printing. 3D printed scaffolds showed good shape accuracy, and SEM and XRD analyses revealed a homogeneous and amorphous structure. Moreover, scaffolds were stable and kept their shape and size after a cycle of compression sweeps. Their integrity was also maintained after immersion in PBS at 37 °C, showing a high swelling capacity, suitable for exudate absorption in wound healing applications.

## 1. Introduction

Natural polymers are commonly used for biomedical purposes due to their high biocompatibility, biodegradability, and bioactivity [1]. In this sense, hydrogels from natural polymers for 3D bioprinting are gaining notoriety to produce scaffolds for applications such as wound dressing [2]. In particular, polysaccharides and proteins are applied as bioinks for 3D printing [3,4,5]. Among them, chitosan has caught attention due to its antimicrobial activity [6,7]. Chitosan is a linear polysaccharide derived from chitin, found in the exoskeleton of crustaceans and in fungi [8]. Furthermore, due to its positive charge in acidic media, it is the only natural cationic polysaccharide. Thus, it has the ability to interact with negatively charged biomolecules by electrostatic interactions that affect mucoadhesion, hemostatic activity, antimicrobial activity, cell permeation capacity, and cytocompatibility [9].

Chitosan has been studied for different biomedical applications, including drug delivery and tissue engineering [10,11,12,13]. However, chitosan presents challenges in 3D printing, since it is too soft to self-support its structure and would collapse or deform due to its own weight [14,15]. Consequently, in order to overcome these drawbacks, chitosan can be combined with other polymers to promote physical and/or chemical crosslinking [8,16]. Although physically crosslinked materials show lower mechanical strength compared to chemically crosslinked ones, physically crosslinked hydrogels exhibit shear thinning behavior and facilitate flow through the needle of the 3D printer [17]. In this regard, chitosan bioinks reinforced with other biopolymer such as gelatin have been successfully printed, controlling the stability of the scaffolds as a function of the gelatin content [18].

In this work, pectin has been selected to reinforce chitosan hydrogels. Pectin is an anionic heteropolysaccharide with gelling properties, extracted from citrus fruits [19,20]. Depending on the esterification degree, pectin can be classified as high or low methoxy pectin [21]. Pectin is highly available, biodegradable, and non-toxic, which makes it a potential material for biomedical applications [22,23]. Moreover, the mixture of chitosan and pectin forms a polyelectrolyte complex mainly conformed by electrostatic interactions between the amino groups of chitosan and the carboxylic groups of pectin [24].

The aim of this work was to prepare chitosan-pectin hydrogels with different concentrations in order to select the optimal composition for 3D printing and analyze the properties of the printed scaffold. To accomplish this challenge, chitosan-pectin hydrogels were assessed from a rheological perspective. The hydrogel that fitted 3D printing requirements was selected, 3D printed, and the resulting scaffolds were physicochemically and mechanically characterized.

## 2. Results and Discussion

### 2.1. Rheological Characterization

#### 2.1.1. Linear Viscoelastic Properties

**Figure 1:**
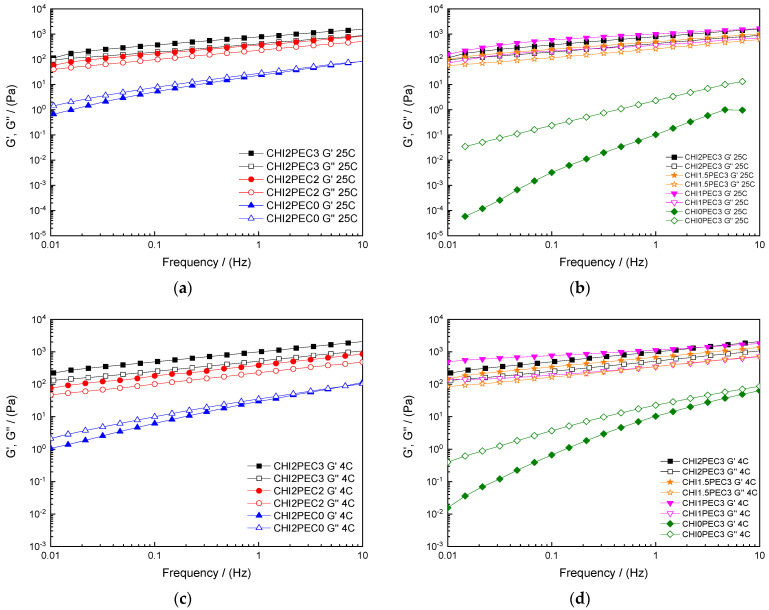
Elastic (G′) and viscous (G″) moduli as a function of frequency from 0.01 to 10 Hz: (**a**,**c**) for chitosan-pectin hydrogels with 2% chitosan at 25 and 4 °C, respectively; (**b**,**d**) for chitosan-pectin hydrogels with 3% pectin at 25 and 4 °C, respectively.

Rheological properties of chitosan-pectin systems were characterized to determine the most suitable hydrogel composition for 3D printing. Frequency sweep tests were performed at 4 and 25 °C to confirm chitosan-pectin gel formation and observe the time dependence of the systems (Figure 1). Regardless of the temperature used during the test (25 or 4 °C), the single system containing only chitosan (CHI2PEC0) showed a predominantly viscous response with the loss modulus (G″) greater than storage modulus (G′) in the low frequency regime (Figure 1a,c) and a crossover point at high frequency (i.e., at 8.60 Hz and 6.45 Hz for 25 °C and 4 °C, respectively). This difference in the crossover frequency suggests that the CHI2PEC0 system needed longer relaxation times at lower temperatures, as a consequence of a slightly more elastic behavior [1]. This behavior is qualitatively similar to that one previously found for single CHI solutions [25]. However, the values of G′ and G″ were higher in the present study since the molecular weight was almost twice the value used in the previous study. In contrast, CHI0PEC3 systems presented a predominantly viscous behavior (G″ > G′) in the whole frequency range (Figure 1b,d), indicating a liquid-like behavior at both temperatures. On the other hand, binary systems containing both biopolymers, chitosan, and pectin (Figure 1a–d), showed a weak gel-like behavior, with G′ values higher than G″, regardless of the frequency and temperature analyzed. The similar and moderate frequency dependence shown by both moduli, for every chitosan/pectin system is typical of weak gels with non-covalent bonding among the components of the formulation [26]. It is worth noting that a gel-like behavior typically results in proper stability to maintain dimensional firmness during deposition on the printing bed [27].

In this sense, parameters *a* and *b* from the power law model for G′ (Equation (1)) were used to predict gel firmness. The power law coefficient, *a*, represents the magnitude of G′ at a frequency of 1 Hz, and the power law exponent, *b*, indicates the dependence of the elastic modulus on frequency, where an exponent value equal to zero means that G′ does not depend on frequency [28]. Power law parameters are shown in Table 1 as a function of the hydrogel composition and temperature. As may be observed, an increase in pectin content led to an increase in parameter *a* giving rise to an enhancement of the gel strength, either at 25 or 4 °C. Moreover, a high level of gel strength was maintained at the highest pectin content even if the chitosan content was reduced from 2 to 1%. As for parameter *b*, results revealed that when pectin concentration increased and chitosan concentration decreased, *b* parameter decreased, resulting in less frequency-dependent hydrogels. Therefore, the increase of pectin led to a stronger hydrogel [29]. Moreover, comparing the same hydrogels at different temperatures, 4 and 25 °C, no significant differences (*p* > 0.05) were generally observed for parameters *a* and *b*. The exceptions were found at the lowest concentrations of chitosan (CHI1PEC3 and CHI0PEC3) that showed lower values for parameter *b* at low temperature. In any case, parameter *b* ranged between 0.18 and 0.36, indicating a relatively marked frequency dependence. This response was reported to correspond to a weak gel behavior [30].

In order to determine the linear viscoelastic range (LVR), stress sweep tests at 25 °C and 4 °C were carried out, which provide information on the unperturbed structure of the system. To delimit the LVR, the critical strain ($\gamma_{c}$) was determined, and values are shown in Table 2. Results revealed significant differences (*p* < 0.05) between single and binary systems, since a decrease of critical strain was observed for the latter, suggesting that the hydrogel network had lower deformation capacity due to the interactions between chitosan and pectin [31]. Chitosan may act as an efficient cross-linker in pectin systems at acidic pH, promoting an upward evolution in the viscoelastic moduli of samples [32]. Additionally, electrostatically stabilized complexes are formed in the presence of both biopolymers, where also hydrogen bonding and hydrophobic interactions may play a role [33].

In addition, regarding G′ values at 1 Hz (G′_1_) shown in Table 3 for the LVR, significant differences (*p* < 0.05) were observed for the hydrogels tested at 25 °C. Thus, the single systems (CHI0PEC3 and CHI2PEC0) showed the lowest G′ values, between 0.11 and 22.96 Pa, respectively. No significant (*p* > 0.05) differences were observed between G′_1_ values at 25 °C and 4 °C for CHI0PEC3, CHI2PEC0, and CHI2PEC2 hydrogels. However, G′_1_ for CHI1PEC3, CHI1.5PEC3, and CHI2PEC3 samples showed a significant increase (*p* < 0.05) with increasing temperature from 4 °C to 25 °C, especially for CHI1PEC3 hydrogels. Therefore, an increase in hydrogel stiffness was driven by a higher proportion of pectin [34].

The predominantly viscous response observed for CHI0PEC3 and CHI2PEC0 systems can be confirmed with the values of tan δ at 1 Hz (tan δ_1_), which are higher than unity for these two systems at 25 °C and 4 °C. In contrast, all the binary chitosan-pectin hydrogels presented tan δ_1_ values lower than 1, indicative of a predominantly elastic behavior. Among chitosan-pectin hydrogels, no significant difference (*p* > 0.05) was observed for tan δ_1_, displaying values around 0.5. Therefore, considering that hydrogels exhibited small critical strain values (γ_c_ < 0.05), moderately low loss tangent values (tan δ_1_ > 0.1), chitosan-pectin hydrogels can be considered “weak gels” [30]. It is worth mentioning that these gels have the capacity to flow without fracture, recover their structure, and achieve the properties required for 3D printing [35].

**Figure 2:**
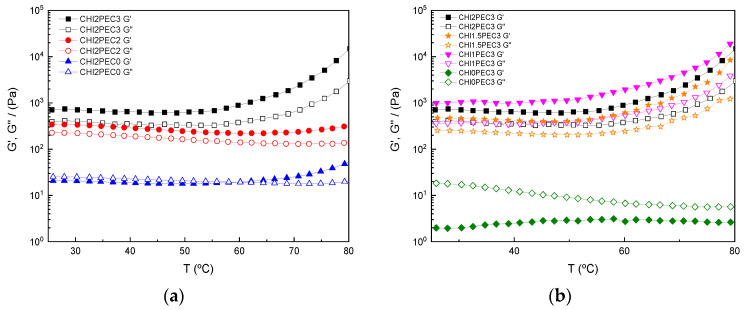
Elastic (G′) and viscous (G″) moduli as a function of temperature from 25 to 80 °C for (**a**) chitosan-pectin hydrogels with 2% chitosan and (**b**) chitosan-pectin hydrogels with 3% pectin.

Temperature sweep tests from 25 to 80 °C were carried out at 1 Hz to analyze the effect of temperature on the rheological behavior of the hydrogels. As shown in Figure 2a, G″ values were higher than G′ for chitosan hydrogels without pectin (CHI2PEC0) at low temperatures, indicating a fluid-like viscoelastic behavior until the crossover point was reached around 58 °C. From that temperature on, hydrogels exhibited a predominantly elastic response (i.e., G′ became higher than G″), due to the formation of chitosan clusters by hydrophobic interactions [36]. In contrast, in absence of chitosan, pectin systems (CHI0PEC3) exhibited a viscous behavior, with G″ values clearly higher than G′ in the whole range of temperatures, as shown in Figure 2B. In any case, these two single systems showed mechanical spectra corresponding to polymer solutions below the threshold for the critical gel behavior.

Regarding chitosan-pectin systems (Figure 2a,b), all samples showed higher values of G′ than G″, reflecting the elastic behavior of the samples in the range between 25 and 80 °C. Moreover, all chitosan-pectin systems presented nearly constant values of both moduli between 25 and 58 °C, suggesting that no difference was expected when printing at room or physiological temperature. However, for temperatures above 58 °C, all chitosan-pectin hydrogels, except CHI2PEC2, presented an increase of both moduli until 80 °C.

#### 2.1.2. Flow Properties

**Figure 3:**
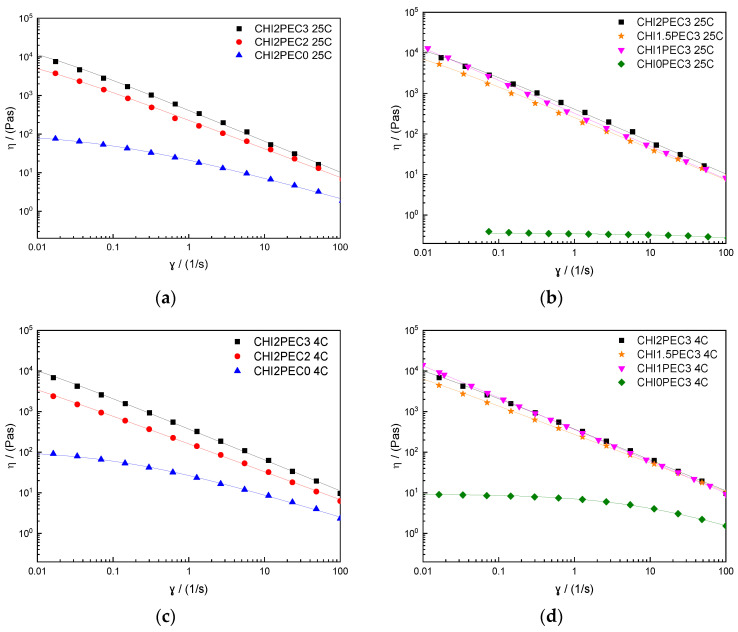
Steady state flow curves of chitosan-pectin hydrogels: (**a**,**c**) for chitosan-pectin hydrogels with 2% chitosan at 25 and 4 °C, respectively; (**b**,**d**) for chitosan-pectin hydrogels with 3% pectin. Lines reproduce the fitting to the Williamson model.

**Figure 4:**
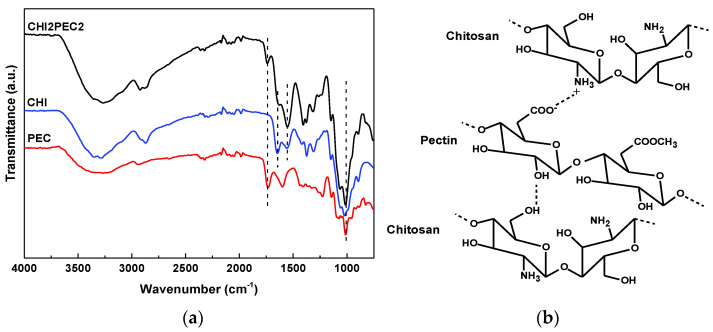
(**a**) FTIR spectra of neat chitosan (CHI), neat pectin (PEC) and the hydrogel with 2 wt% chitosan and 2 wt% pectin (CHI2PEC2) and (**b**) representation of the interactions between chitosan and pectin.

Additionally, the flow behavior of chitosan-pectin hydrogels was determined, and flow curves are displayed in Figure 3. All chitosan-pectin hydrogels with 2% chitosan presented a similar shear-thinning behavior at 25 °C (Figure 3a) and 4 °C (Figure 3c), with a marked decrease of viscosity as shear rate increased, clearly describing a power law decay region and showing a tendency towards a zero-shear limiting viscosity at low shear rate. Once again, the single CHI solution (CHI2PEC0) displayed a similar shear thinning response, although showing higher viscosity values than those found previously (due to the highest molecular weight) [25]. Moreover, flow properties were dependent on pectin concentration; when pectin concentration increased, viscosity also increased. However, binary systems containing 3% pectin did not reflect any apparent influence of chitosan concentration on the flow curves, neither at 25 °C (Figure 3b) nor at 4 °C (Figure 3d). In contrast, pectin hydrogels without chitosan were found to be independent on shear rate at 25 °C, indicating a Newtonian fluid behavior, whereas at 4 °C, a decrease of viscosity was observed at high shear rates, indicating a non-Newtonian shear-thinning behavior. Moreover, the viscosity at 4 °C increased 10 times with respect to the viscosity at 25 °C, as also reported by other authors [37,38]. It is worth mentioning that the shear thinning behavior of the hydrogels facilitates their flow during 3D printing. Upon hydrogel ejection from the nozzle of the 3D printer (25 °C) and its deposition on the printer bed (4 °C), the shear rate undergoes a sudden decrease which entails a remarkable increase in the viscosity of the shear-thinning hydrogel, leading to an enhancement of the dimensional stability of the 3D printed scaffold [39].

Flow curves were fitted to the Williamson model (Equation (2)) and fitting parameters are presented in Table 4. Concerning the zero-shear rate viscosities for the hydrogels at 25 °C, an increase from 19.7 to 1523 kPa·s was observed following this increasing sequence: CHI2PEC2 < CHI2PEC3 < CHI1.5PEC3 < CHI1PEC3, indicating that when the amount of pectin increased and that of chitosan decreased, the formation of polymer aggregates was promoted [40,41]. Regarding the effect of temperature, no significant difference (*p* < 0.05) was observed between the values of the zero-shear rate viscosity at 25 and 4 °C. Regarding the viscosity in the 3D printing process, taking into consideration that the nozzle diameter used was 0.84 mm, with a printing velocity of 2.8 mm/s, the shear rate can be calculated using Equation (3). The estimated value for the shear rate at the nozzle was between 42 and 60 s^−1^, corresponding to a viscosity between 12 and 23 Pa·s for all binary systems. Bearing in mind these results, viscosity during 3D printing decreased 10^3^ times compared to the zero-shear rate viscosity. These results are in accordance with the empirical ink viscosities reported during 3D printing, which are in a range from 0.5 to 20 Pa·s at high shear rates (10^2^–10^3^ s^−1^), while the range moves from 10^2^ to 10^3^ Pa·s at lower shear rates (≤0.1 s^−1^) [42,43,44]. While all binary systems follow the criteria for high shear rates, only CHI2PEC2 and CHI1.5PEC3 binary systems comply with the requirement at low shear rates (i.e., viscosity lower than 10^3^ Pa·s). In order to select the most suitable system for 3D printing applications, the following features can be considered: (i) Systems containing 3% pectin showed apparent viscosities higher than 1000 Pa·s at low shear rates (<0.1 s^−1^); (ii) Higher concentrations of pectin with lower concentrations of chitosan showed pectin aggregation and clustering and, thus, heterogeneity; (iii) There was no significant difference in the rheological behavior among the rest of chitosan-pectin hydrogels. Therefore, taking all these factors into account, CHI2PEC2 hydrogels were selected for 3D printing.

### 2.2. CHI2PEC2 Hydrogel Characterization

The FTIR spectra of chitosan, pectin, and CHI2PEC2 are shown in Figure 4a. Both chitosan and pectin showed bands at 3000–3600 cm^−1^, associated with O-H bonds in both polymers and to N-H bonds in chitosan, and bands between 1150 and 890 cm^−1^ associated with the C-O-C of the saccharide ring [33]. The differences were observed in the range between 1300 and 1750 cm^−1^. Regarding chitosan spectrum, the band at 1644 cm^−1^, attributed to C=O stretching; a band at 1558 cm^−1^, associated to NH2 bending; and two bands at 1418 and 1376 cm^−1^, corresponding to CH3 deformation, were observed [45,46]. For pectin spectrum, a band at 1738 cm^−1^, assigned to C = O of the ester bonds, and the band at 1604 cm^−1^, associated with the asymmetric stretching vibration of COO- were observed [47]. Some displacements of these bands were observed for CHI2PEC2. In particular, the band related to the ester group in pectin shifted from 1738 cm^−1^ to 1732 cm^−1^ in CHI2PEC2. Moreover, the absorption band corresponding to C=O stretching at 1644 cm^−1^ for chitosan and at 1604 cm^−1^ for pectin shifted to 1628 cm^−1^ for CHI2PEC2. All these band displacements indicate physical interactions by hydrogen bonding between both biopolymers (Figure 4b).

**Figure 5:**
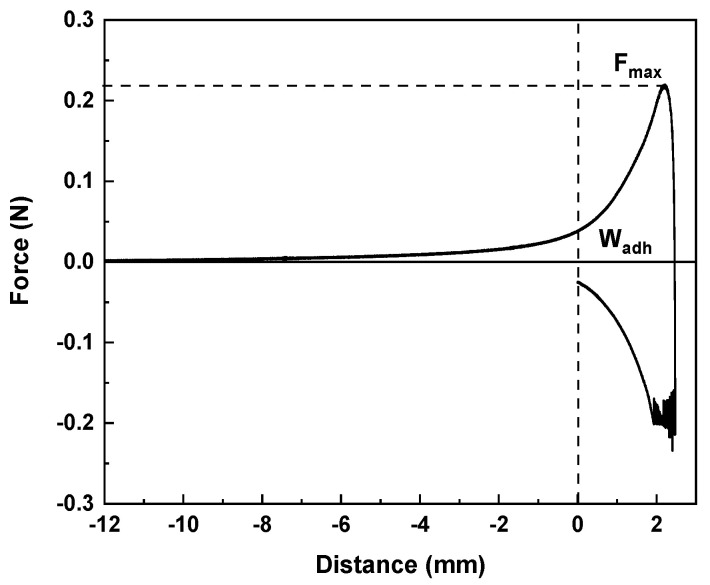
Force displacement curve of one replicate of CHI2PEC2 hydrogel in the mucoadhesion analysis.

In order to evaluate the hydrogel adhesiveness to a biological surface, mucoadhesion analysis was carried out and the force displacement curve of one of the CHI2PEC2 replicates is shown in Figure 5. At the beginning of the test, when the sample is getting closer to the mucin disk, the force decreased due to the force applied to attach the hydrogel to the mucin disk. A significant increase of the force was observed as the hydrogel started to detach from the mucin disk due to the exerted force done. From then on, the force started to decrease. The maximum detachment force (Fmax) and the work of adhesion (Wad) were calculated from the mucoadhesion analysis. The values found for Fmax and Wad were 0.21 ± 0.02 N and 0.36 ± 0.03 N mm, respectively. These values are higher than those obtained for other polysaccharide systems (Fmax = 0.093 N and Wad = 0.029 N mm) for wound dressing applications [48]. Therefore, chitosan-pectin hydrogel showed excellent mucoadhesive properties, which are essential requirements for wound dressing materials in order to be adhered to the wound site to protect it from the external environment. This mucoadhesive capacity is associated with –OH and –NH2 groups in the biopolymers, which could be linked to mucins by hydrogen bonding; furthermore, as acidic pH was used for the hydrogel preparation, the amino groups of chitosan were protonated and, thus, strong electrostatic interactions could be formed with the anionic groups of mucin [49]. Since pectin and mucin are anionic compounds, hence, electrostatic repulsion charges might result in an uncoiling of polymer chains and facilitate chain entanglement and bond formation [50].

Additionally, texture profile analysis (TPA) can provide information related to the hardness, adhesiveness, and cohesiveness of the hydrogels, which are of great relevance to analyze their handleability for 3D printing [51]. In this regard, hardness, which is the maximum force required to produce the first deformation, showed a value of 5.9 ± 0.2 g. Adhesiveness is related to the capacity of a gel to adhere on a surface and, thus, higher adhesiveness values indicate that the gel needs shorter time to bond to the surface [52]. In the case of the CHI2PEC2 system, the adhesiveness value found was 8.7 ± 0.5 g·s. This adhesiveness, as well as the hardness observed for CHI2PEC2 hydrogel, was similar to values obtained for hyaluronic acid/carboxymethylcellulose systems [50]. Finally, cohesiveness is related to the strength capacity of a gel to maintain its own structure when subjected to a compressive stress [53]. CHI2PEC2 hydrogel exhibited a high cohesive value of 1.05 ± 0.04, indicative of a high capacity to maintain the three-dimensional structural integrity and, hence, to hold its structure after printing [54].

### 2.3. 3D Printed CHI2PEC2 Scaffold Characterization

**Figure 6:**
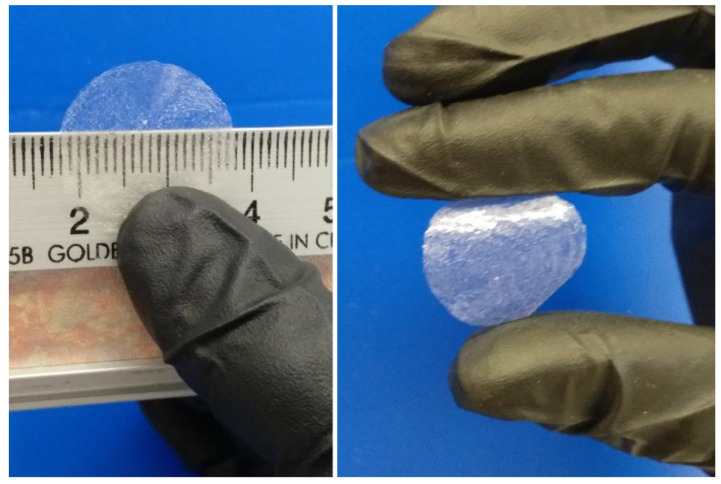
3D printed CHI2PEC2 scaffold after drying at room temperature.

CHI2PEC2 scaffolds were successfully printed with shape fidelity as shown in Figure 6. After ink deposition, the material kept the set geometry and showed mechanical integrity.

#### 2.3.1. Physicochemical Properties

**Figure 7:**
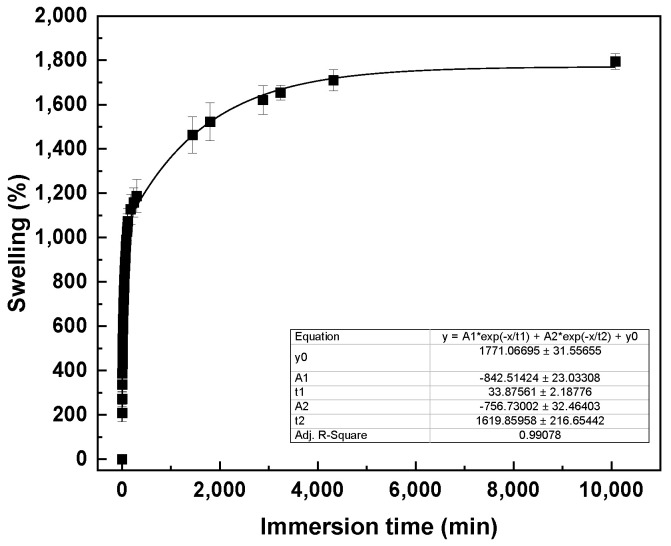
Swelling capacity of CHI2PEC2 scaffolds in PBS (pH = 7.4).

Swelling of CHI2PEC2 scaffolds was evaluated in phosphate buffered saline (PBS) solution (pH = 7.4) at room temperature. As can be observed in Figure 7, swelling increased fast up to 1250% in the first 5 h, indicating that the samples increased more than 10 times their initial weights. Thereafter, swelling continued increasing more slowly up to 1830% at day 7. Since pectin moieties were negatively charged with -COO- groups at pH 7.4, electrostatic repulsions were promoted and, thus, the swelling ability increased [55,56]. In this context, having a high swelling capacity is desirable in order to absorb wound exudates that could slow the wound healing and macerate the nearby skin [57]. It is worth noting that samples kept their integrity after the 7 days of immersion.

**Figure 8:**
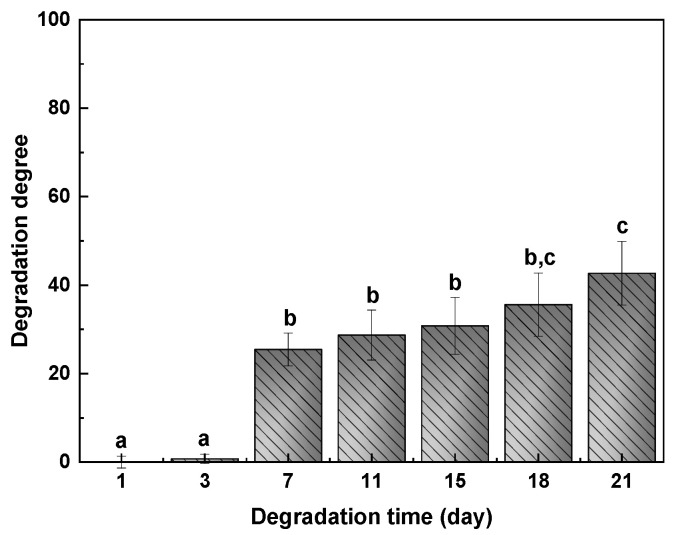
Degradation degree of CHI2PEC2 scaffolds immersed into PBS at 37 °C. ^a–c^ Two bars with the same letter are not significantly (*p* > 0.05) different according to the Tukey’s multiple range test.

The values of degradation degree (DD) after 1, 3, 7, 11, 15, 18, and 21 days of immersion into PBS at 37 °C are presented in Figure 8. No significant mass loss (*p* > 0.05) was observed up to day 7. After 7 days, a pronounced increase of DD up to 25% was observed, but samples kept their integrity, which indicates that the interactions between chitosan and pectin were strong enough. No significant differences (*p* > 0.05) were observed between days 7 and 18, at which an increase of DD and a mass loss of 43% were observed.

#### 2.3.2. Scaffold Structure and Mechanical Properties

**Figure 9:**
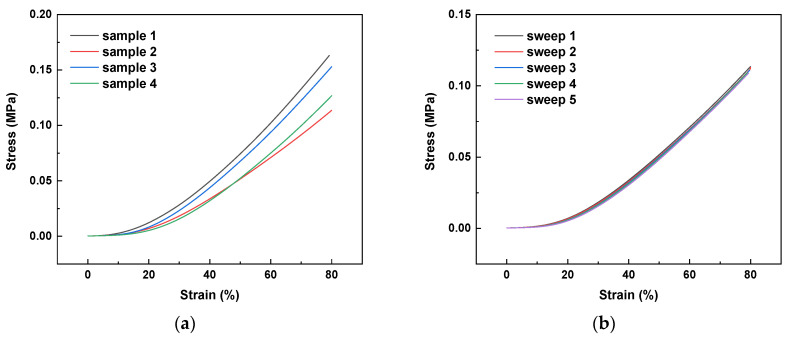
Stress-strain curves: (**a**) for four samples of CHI2PEC2 scaffolds; (**b**) for sample 2 subjected to a cycle of five compression sweeps.

Since the hydrogel will be hydrated in the in vivo milieu, the scaffolds were hydrated in a 100% humidity environment for 48 h before the test was carried out. Stress-strain curves for CHI2PEC2 samples are shown in Figure 9a. As can be observed, a linear elastic behavior is observed up to 80% of strain. There was no significant difference (*p* > 0.05) among different samples, and an average stress of 0.002 ± 0.001, 0.008 ± 0.003, and 0.14 ± 0.02 MPa was determined for a strain of 10%, 20%, and 80%, respectively. Furthermore, as can be observed for sample 2 in Figure 9b, scaffolds maintained their shape and size after a cycle of five compression sweeps.

**Figure 10:**
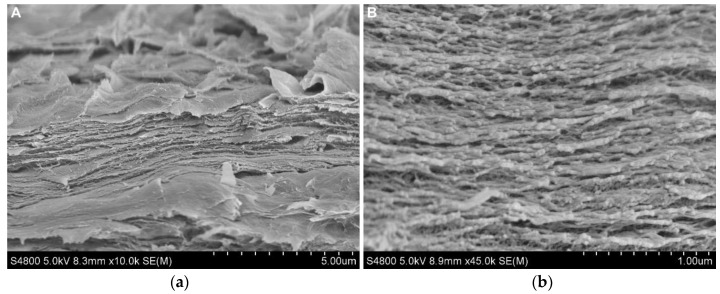
SEM images of CHI2PEC2 cross-section: (**a**) ×1000 magnification and (**b**) ×4500 magnification.

SEM analysis was performed in order to evaluate the scaffold morphology. SEM images are shown in Figure 10, where cross-sections with a magnification of ×1000 and ×4500 are presented. As can be observed, the scaffold showed a homogeneous structure in which different layers can be differentiated.

**Figure 11:**
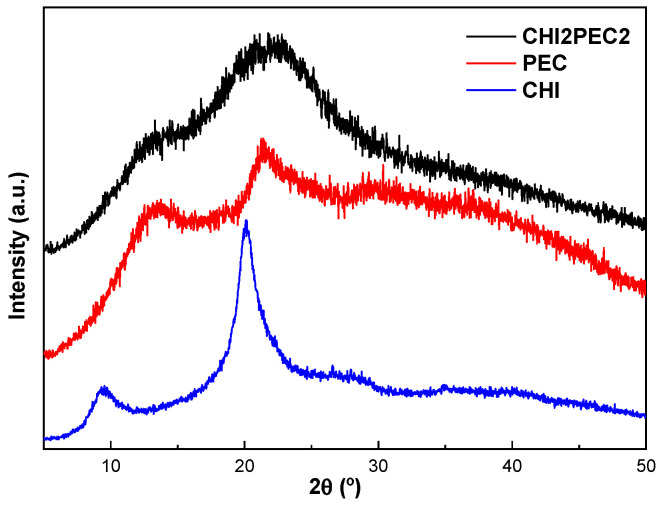
XRD diffractograms of neat chitosan (CHI), neat pectin (PEC), and CHI2PEC2 scaffold.

Additionally, XRD measurements plotted in Figure 11 showed the two characteristic peaks of chitosan at 9.5° and 20.1°, and three peaks at 13.5°, 21.3° and 30° for pectin, all of them related to the semicrystalline structure of these polysaccharides [58]. Concerning the CHI2PEC2 scaffold, two broad peaks were observed at 13.5° and 21.8°, revealing an amorphous structure due to the intermolecular hydrogen bonding and electrostatic interactions between chitosan and pectin [59].

## 3. Conclusions

Rheological analysis of hydrogels with different concentrations of chitosan and pectin was performed in order to select the optimal composition to be 3D printed and obtain dimensionally stable scaffolds. Chitosan-pectin systems showed a weak gel behavior with shear thinning flow properties. Furthermore, they showed favorable properties for 3D printing, keeping geometry and mechanical integrity after being printed at room or physiological temperatures. However, systems with a high concentration of pectin presented aggregation; therefore, CHI2PEC2 hydrogel was selected as the optimal system for 3D printing. CHI2PEC2 exhibited physical interactions between chitosan and pectin, as shown by FTIR analysis, and high cohesiveness, related to the capacity of keeping shape and size after 3D printing. Likewise, high mucoadhesiveness was observed and related to the hydrogen bonding and the electrostatic interactions of chitosan and pectin with mucin. The CHI2PEC2 scaffold presents a homogeneous morphology, as shown by the SEM images of cross-sections, and an amorphous structure revealed by XRD analysis. Furthermore, the scaffold exhibited good properties for biomedical applications such as wound dressing since it showed a high swelling capacity, suitable for wound exudate absorption, and high strength to maintain shape and size after a cycle of compression sweeps.

## 4. Materials and Methods

### 4.1. Materials

Chitosan, with a molecular weight of 375 kDa and a deacetylation degree ≥75%, was supplied by Sigma-Aldrich, Spain. High methoxylated pectin, with a molecular weight of 472 kDa and an esterification degree of 58%, was kindly supplied by CEAMSA, Spain. Acetic acid solution (1 N), used as solvent, was supplied by Panreac, Spain.

### 4.2. Hydrogel Preparation

Chitosan-pectin systems were obtained by mixing chitosan and pectin solutions, previously prepared. On the one hand, the required amount of pectin was dissolved in water by stirring at 67 °C to obtain a 3% (*w*/*v*) aqueous solution. This solution was left at 4 °C for 24 h before any further processing or characterization. This system was designated as CHI0PEC3. On the other hand, a certain amount of chitosan was dissolved in a 1% acetic acid aqueous solution for 30 min under stirring to obtain a 2% (*w*/*v*) chitosan solution designated as CHI2PEC0. Then, the corresponding volumes of both solutions were mixed at 7000 rpm for 10 min (Ultraturrax UT25, IKA, Staufen, Germany) to achieve the binary systems depicted in Table 4. The resulting binary systems were stored at 4 °C for 24 h, at least, until further processing or characterization.

### 4.3. Rheological Characterization

A Haake MARS rheometer (Thermo Fisher Scientific, Vigo, Spain) coupled with a Universal Temperature Control (UTC) unit (Thermo-Scientific, Vigo, Spain) was used for rheological characterization. The geometry used was a serrated plate–plate geometry, with a diameter of 35 or 60 mm, depending on the consistency of the system, and a gap between plates of 1 mm.

Stress sweep tests were initially carried out from 0.01 Pa to 1000 Pa at a constant frequency (1 Hz) to determine the critical stress and critical strain that define the linear viscoelastic range (LVR) of the systems. Then, frequency sweep tests were performed from 0.01 to 10 Hz within the LVR of each system. Elastic modulus (G′), viscous modulus (G″), and loss tangent (tan δ) were obtained. A power law correlation between G′ and the frequency was calculated using Equation (1) to model the frequency dependence of G′.

(1) $$\;G^{\prime }=a\cdot \;\omega^{b},$$

where *ω* is the frequency, *a* is a coefficient that represents the magnitude of G′ at a frequency of 1 Hz, and *b* is a power law exponent that describes the dependence of the slope of G′ on frequency.

Both dynamic tests were performed at two different temperatures (25 °C and 4 °C) to simulate the potential behavior of the systems studied during extrusion and on the 3D printer bed after deposition.

Regarding temperature sweep tests from 25 to 80 °C, a constant frequency of 1 Hz and a heating rate of 3 °C/min were set. Thermal treatments were always performed within the LVR, as stress was kept constant at a stress lower than the critical stress determined.

Shear flow tests were accomplished with a step-by-step increase of the shear rate ($\dot{\gamma }$) over the range 0.01–100 s^−1^, and the steady state was obtained at each shear rate. Flow data were fitted to the model developed by Williamson for shear-thinning materials (Equation (2)):

(2) $$\eta =\frac{\eta_{0}}{1+{\left(\lambda \dot{\gamma }\right)}^{1-n}},$$

where *η*_0_ is the zero-shear rate viscosity, λ is a characteristic time for the onset of the shear-thinning region, and 1 − *n* is the slope of this region. The parameter n represents the flow index of the power law region.

Moreover, an empirical viscosity during 3D printing was determined calculating the maximum shear rate at the nozzle using the Weissenberg-Rabinowitsch-Mooney equation (Equation (3)), assuming that printing takes place at shear rates within the power law region

(3) $${\dot{\gamma }}_{W}=\frac{8\;V}{D}\cdot \left(\frac{3n+1}{4n}\right),$$

where *V* is the 3D printing velocity, *D* is the nozzle diameter, and *n* is the flow index.

Once the gap was achieved, all samples were left for 5 min before running the test to allow residual stress to relax and to stabilize sample temperature. At least three replicates were tested for each system.

### 4.4. Characterization of CHI2PEC2 Hydrogel

#### 4.4.1. Fourier Transform Infrared (FTIR) Spectroscopy

FTIR spectra were performed with a Nicolet Nexus FTIR spectrometer (Thermo Fisher Scientific, Madrid, Spain) with a Golden Gate ATR accessory. Spectra were collected with 32 scans with a resolution of 4 cm^−1^ in the wavelength between 4000 and 800 cm^−1^.

#### 4.4.2. Mucoadhesion Study

Mucoadhesive properties were determined by means of a TA.XT.Plus Texture Analyzer equipped with a 5 kg load cell, a gel mucoadhesion accessory (A/GMP), and a mucoadhesion rig (A/MUC). The maximum force required to separate the hydrogel from the membrane (maximum detachment force) and the total amount of force involved in the film withdrawal from the membrane (work of adhesion) were calculated by triplicate with a disc soaked in 1% porcine stomach’s mucin in a phosphate buffered saline (PBS) at 37 °C.

#### 4.4.3. Texture Profile Analysis (TPA)

Texture profile analysis of hydrogel was carried out with a TA-XT plus Texture Analyzer equipped with a 5 kg load cell and an aluminum cylinder of 50 mm diameter (P/50). Samples were compressed twice until 20% of the original height and with a delay time of 5 s and activation force of 0.1 g. The operating speed was set at 1 mm/s and three replicates were collected for CHI2PEC2 hydrogel. Hardness, adhesiveness, and cohesion were determined by using the software Exponent 8,0,5,0.

### 4.5. 3D Printing of CHI2PEC2 Hydrogel and Scaffold Characterization

CHI2PEC2 scaffolds were printed using a syringe-based extrusion 3D DomoBIO printer (Domotek, Tolosa, Spain). The scaffold design, a cylindrical mesh with 21 mm of diameter, 1.5 mm height, and infill of 100%, was accomplished with Cura (Ultimaker Cura 4.6.1) software. A syringe with the ink was placed into the cartridge with the printing temperature fixed at 25 °C. The syringe was placed in the cartridge at 25 °C for 30 min before printing to have a homogeneous temperature distribution. The hydrogel was printed in the following conditions: G18 nozzle (inside diameter of 0.84 mm), dosing distance of 0.17 mm, 2.8 mm/s printing speed, and 120 mm/s non-printing speed. After printing, the scaffold was dried by evaporation of the solvent at room temperature.

#### 4.5.1. Swelling Measurements

In order to perform the swelling test, a gravimetric method was followed. Printed samples were weighed (*W*_0_) and subsequently immersed into 70 mL of PBS (pH = 7.4). After immersion the samples were weighed (*W_h_*) every 2 min for the first hour, then every 10 min for the second hour and every hour for the next 3 h. Thereafter, measurements were carried out in triplicate at 24 h, 30 h, 48 h, 54 h, 72 h, and 7 days. The swelling degree (*S*) was calculated by Equation (4):

(4) $$S\;\left(\%\right)=\frac{W_{h}-W_{0}}{W_{0}}100,$$

#### 4.5.2. Degradation Degree (*DD*)

Degradation degree (*DD*) was performed with scaffolds (*W*_0_) immersed in PBS (pH 7.4) at 37 °C for days 1, 3, 7, 11, 15, 18, and 21 (*W_d_*). After immersion, samples were left to dry until reaching constant weight. Three replicates were tested, and *DD* was calculated as follows:

(5) $$DD\;\left(\%\right)=\frac{W_{0}-W_{d}}{W_{0}}100.$$

#### 4.5.3. Compression Test

CHI2PEC2 printed scaffolds were compressed using a TA.XT plusC Texture Analyzer equipped with a 5 kg load cell and an aluminum cylinder of 50 mm diameter (P/50). Before testing, samples were kept in a chamber at room temperature and 100% relative humidity for 48 h. A cyclic compression test was performed with five sweeps since scaffolds recovered their initial height at the end of the compression. One mm/s crosshead speed and activation force of 5 g were set as compression conditions. The test was carried out at room temperature and the load was applied until 80% of the scaffold initial height was compressed. Data were analyzed with Exponent 8,0,5,0 software.

#### 4.5.4. Scanning Electron Microscopy (SEM)

A Hitachi S-4800 scanning electron microscope (Hitachi High-Technologies Corporation, Tokyo, Japan) with an acceleration voltage of 15 kV was used to visualize the scaffold cross-section morphologies. The sample was placed in a metallic stub and covered with gold under vacuum in argon atmosphere.

#### 4.5.5. X-ray Diffraction (XRD)

X-ray diffraction (XRD) measurements were performed with a PANalytic Xpert Pro (PANalytical, Almelo, The Netherlands) equipped with a diffraction unit and Cu-Kα (λ= 1.5418 Å) as a radiation source. Analysis was set at 40 kV and 40 mA, and data were collected from 2° to 40° (step size = 0.026, time per step = 118 s).

### 4.6. Statistical Analysis

With the purpose of determining the significant differences between measurements, analysis of variance (ANOVA) was carried out by means of SPSS software (SPSS Statistic 26). Tukey’s multiple range test was used for multiple comparisons among different systems with a statistical significance at the *p* < 0.05 level.

## Figures and Tables

**Table 1:** The effect of chitosan and pectin concentration on *a* coefficient and *b* exponent of the power law model for the storage modulus in the frequency sweep of the hydrogels at 25 and 4 °C.

T (°C)	Sample	*a* (Pa·Hz ^b^)	*b*	R^2^
25	CHI2PEC0	20.1 ± 1.1 ^a,A^	0.677 ± 0.015 ^a,A^	0.9922
	CHI2PEC2	340 ± 19 ^b,B^	0.360 ± 0.008 ^b,B^	0.9992
	CHI2PEC3	759 ± 3 ^c,C^	0.328 ± 0.001 ^b,BC^	0.9980
	CHI1.5PEC3	485 ± 18 ^b,B^	0.300 ± 0.011 ^bc,C^	0.9992
	CHI1PEC3	725 ± 130 ^c,C^	0.236 ± 0.013 ^c,D^	0.9977
	CHI0PEC3	0.0938 ± 0.0002 ^a,A^	1.580 ± 0.059 ^d,E^	0.9858
4	CHI2PEC0	26.9 ± 0.4 ^A^	0.676 ± 0.001 ^A^	0.9959
	CHI2PEC2	381 ± 4 ^B^	0.346 ± 0.003 ^BC^	0.9991
	CHI2PEC3	1233 ± 37 ^D^	0.303 ± 0.011 ^BC^	0.9998
	CHI1.5PEC3	753 ± 81 ^C^	0.295 ± 0.011 ^C^	0.9996
	CHI1PEC3	1209 ± 68 ^D^	0.176 ± 0.005 ^F^	0.9965
	CHI0PEC3	8.90 ± 0.49 ^A^	1.125 ± 0.022 ^G^	0.9835

^a–c, A–G^ Two means followed by the same letter in the same column are not significantly (*p* > 0.05) different according to the Tukey’s multiple range test.

**Table 2:** Critical strain (γ_c_), and values for G′ at 1 Hz (G′_1_), and tan δ at 1 Hz (tan δ_1_) within the linear viscoelastic range for the hydrogels tested at 25 °C and 4 °C.

T (°C)	Sample	γ_c_	G′_1_ (Pa)	tan δ_1_
25	CHI2PEC0	0.392 ± 0.016 ^a,A^	23.0 ± 1.4 ^a,A^	1.20 ± 0.03 ^a,A^
	CHI2PEC2	0.034 ± 0.002 ^b,B^	257 ± 18 ^b,B^	0.64 ± 0.03 ^a,AB^
	CHI2PEC3	0.037 ± 0.004 ^b,B^	763 ± 41 ^c,F^	0.54 ± 0.01 ^a,AB^
	CHI1.5PEC3	0.033 ± 0.001 ^b,B^	486 ± 21 ^d,CD^	0.53 ± 0.04 ^a,AB^
	CHI1PEC3	0.025 ± 0.001 ^b,B^	599 ± 31 ^e,DE^	0.39 ± 0.04 ^a,B^
	CHI0PEC3	0.752 ± 0.137 ^c,C^	0.114 ± 0.010 ^a,A^	21.52 ± 0.92 ^b,C^
4	CHI2PEC0	0.333 ± 0.006 ^A^	29.4 ± 0.3 ^A^	1.201 ± 0.016^A^
	CHI2PEC2	0.0243 ± 0.001 ^B^	417 ± 62 ^BC^	0.590 ± 0.003 ^AB^
	CHI2PEC3	0.0380 ± 0.0008 ^B^	1023 ± 52 ^G^	0.505 ± 0.009 ^AB^
	CHI1.5PEC3	0.0218 ± 0.0008 ^B^	660 ± 21 ^EF^	0.498 ± 0.008 ^AB^
	CHI1PEC3	0.0231 ± 0.0004 ^B^	1023 ± 93 ^G^	0.301 ± 0.006 ^B^
	CHI0PEC3	0.295 ± 0.013 ^A^	11.3 ± 1.1 ^A^	2.103 ± 0.085 ^D^

^a–e, A–G^ Two means followed by the same letter in the same column are not significantly (*p* > 0.05) different according to the Tukey’s multiple range test.

**Table 3:** Zero-shear rate viscosity (η_0_), characteristic time (λ), flow index (n), and correlation coefficient (R^2^) of Williamson model for chitosan-pectin hydrogels at 25 and 4 °C.

T (°C)	Sample	η_0_ (Pa·s)	λ (s)	n	R^2^
25	CHI2PEC0	113.84 ± 15.43 ^a,A^	14.2 ± 1.0 ^a,A^	0.448 ± 0.016 ^a,A^	0.999
	CHI2PEC2	19,658 ± 2287 ^a,AB^	431 ± 116 ^b,B^	0.266 ± 0.008 ^b,BCD^	0.999
	CHI2PEC3	29,432 ± 7419 ^a,ABC^	198 ± 74 ^a,AB^	0.191 ± 0.008 ^b,CD^	0.999
	CHI1.5PEC3	36,336 ± 9625 ^a,ABC^	1040 ± 135 ^c,C^	0.265 ± 0.022 ^b,BCD^	0.999
	CHI1PEC3	152,729 ± 31,136 ^b,D^	2621 ± 57 ^d,F^	0.204 ± 0.018 ^b,CD^	0.999
	CHI0PEC3	0.501 ± 0.137 ^a,A^	0.0009 ± 0.0004 ^a,A^	0.674 ± 0.133 ^c,E^	0.970
4	CHI2PEC0	115.71 ± 8.58 ^A^	8.75 ± 0.91 ^A^	0.438 ± 0.013 ^A^	0.999
	CHI2PEC2	37,051 ± 1973 ^ABC^	2632 ± 222 ^F^	0.305 ± 0.007 ^BC^	0.999
	CHI2PEC3	48,159 ± 6715 ^BC^	570 ± 124 ^B^	0.249 ± 0.010 ^CD^	0.999
	CHI1.5PEC3	64,136 ± 7242 ^C^	2131 ± 198 ^E^	0.288 ± 0.007 ^BC^	0.999
	CHI1PEC3	207,319 ± 28281 ^E^	1544 ± 282 ^D^	0.168 ± 0.001 ^D^	0.999
	CHI0PEC3	8.050 ± 1.237 ^A^	0.119 ± 0.028 ^A^	0.374 ± 0.023 ^AB^	0.999

^a–d, A–F^ Two means followed by the same letter in the same column are not significantly (*p* > 0.05) different according to the Tukey’s multiple range test.

**Table 4:** Denotation and composition of chitosan-pectin systems.

	System Designation	Chitosan Concentration (*w*/*v*%)	Pectin Concentration (*w*/*v*%)
Single	CHI2PEC0	2.0	0.0
systems	CHI0PEC3	0.0	3.0
	CHI2PEC2	2.0	2.0
Binary	CHI2PEC3	2.0	3.0
systems	CHI1.5PEC3	1.5	3.0
	CHI1PEC3	1.0	3.0

## Data Availability

Not applicable.
